# Studies on the Application of Polyimidobenzimidazole Based Nanofiber Material as the Separation Membrane of Lithium-Ion Battery

**DOI:** 10.3390/polym15081954

**Published:** 2023-04-20

**Authors:** Yu-Hsiang Lu, Yu-Chang Huang, Yen-Zen Wang, Ko-Shan Ho

**Affiliations:** 1 Department of Chemical and Materials Engineering, National Yu-Lin University of Science & Technology, 123, Sec. 3, University Rd., Douliu 64301, Taiwan; jerrylu34@gmail.com; 2 Department of Chemical and Materials Engineering, National Kaohsiung University of Science and Technology, 415, Chien-Kuo Road, Kaohsiung 80782, Taiwan; ych@nkust.edu.tw

**Keywords:** aromatic polyimide, imidazole polyimide, polybenzimidazole, nano fibered membrane, separator, lithium-ion battery, cyclic stability, rate performance

## Abstract

Aromatic polyimide has good mechanical properties and high-temperature resistance. Based on this, benzimidazole is introduced into the main chain, and its intermolecular (internal) hydrogen bond can increase mechanical and thermal properties and electrolyte wettability. Aromatic dianhydride 4,4′-oxydiphthalic anhydride (ODPA) and benzimidazole-containing diamine 6,6′-bis [2-(4-aminophenyl)benzimidazole] (BAPBI) were synthesized by means of a two-step method. Imidazole polyimide (BI-PI) was used to make a nanofiber membrane separator (NFMS) by electrospinning process, using its high porosity and continuous pore characteristics to reduce the ion diffusion resistance of the NFMS, enhancing the rapid charge and discharge performance. BI-PI has good thermal properties, with a Td5% of 527 °C and a dynamic mechanical analysis Tg of 395 °C. The tensile strength of the NFMS increased from 10.92MPa to 51.15MPa after being hot-pressed. BI-PI has good miscibility with LIB electrolyte, the porosity of the film is 73%, and the electrolyte absorption rate reaches 1454%. That explains the higher ion conductivity (2.02 mS cm^−1^) of NFMS than commercial one (0.105 mS cm^−1^). When applied to LIB, it is found that it has high cyclic stability and excellent rate performance at high current density (2 C). BI-PI (120 Ω) has a lower charge transfer resistance than the commercial separator Celgard H1612 (143 Ω).

## 1. Introduction

In order to mitigate climate change, it is a broad consensus among all countries to use a large amount of renewable energy. However, some renewable energy (e.g., solar and wind energy) has time-intermittent problems [1], so it is necessary to develop a suitable energy storage system for the grid. In this regard, electrochemical energy conversion and storage (EECS) systems are very promising owing to their high turnover efficiencies, fast response times, facile scalability, and freedom from geographical constraints [2]. Lithium-ion batteries’ high energy density and mature manufacturing process make them a popular choice for EECS [3]. Lithium-ion batteries mainly comprise four components, a positive electrode, a negative electrode, a separator, and an electrolyte. Lithium ions move back and forth between the two electrodes during charging and discharging. When charging, lithium ions are released from the positive electrode and transported to the negative electrode through the electrolyte layer. Currently, the negative electrode is in a lithium-rich state, and the positive electrode is in a lithium-poor state. The electronic compensation charge is supplied to the negative electrode through an external circuit to balance the charge [4]. The opposite is true when discharging. It is self-evident that the separator acts as a physical barrier in Li-ion battery devices, keeps the positive and negative electrodes out of direct contact, and houses the electrolyte to facilitate the shuttling of ions within the battery [5,6]. In lithium-ion batteries, the separator is closely related to safety, and the failure of the separator will bring severe risks to the battery [7,8]. A high-performance LIB separator should possess the following characteristics: uniform separator thickness to prevent concentration gradients, maximized porosity with mechanical integrity to enhance electrolyte absorption, and good mechanical properties to prevent contraction when subjected to crimping tension and shrinkage. Good thermal dimensional stability prevents the separator from shrinking at high temperatures, causing internal short circuits. Good electrolyte affinity provides higher rate performance, good chemical/electrochemical stability, etc. [9,10,11]. Currently, commercially available LIB separator materials are mainly polyolefins, which have poor electrolyte affinity, low porosity, and poor capacitance retention under high-power charging and discharging [12]. Unfortunately, polyolefins will shrink at high temperatures [13], which exposes the battery to the risk of thermal runaway [14]. Therefore, it is crucial to develop high-performance and safer separators.

Polyimide has diverse monomer types, simplicity of synthesis, and unique physical and chemical combinations, so it is widely used in many fields [15]. Wu systematically studied polyimides polymerized by four monomers and pointed out that more polar functional groups in the main chain can improve the wettability with LIB electrolytes [16]. Xia. et al. introduced benzimidazole groups into the polyimide backbone [17]. The benzimidazole (C=N: proton acceptor) and amine (-NH-: proton donor) groups can form hydrogen bonding with the carbonyl groups and demonstrate strong intermolecular interaction. The formation of hydrogen bonding is a helpful method for improving polymers’ thermal and mechanical properties [18] by increasing Tg and tensile strength [19].

The electrospinning technique is a process that can stretch a viscous liquid (e.g., polymer solution) into a continuous fiber with a diameter ranging from a few nanometers to a few microns by using an electric field force [20,21,22,23]. Liu. et al. processed polyimide into a nanofiber film using an electrospinning method. They applied it to a LIB to obtain ultra-high porosity, significantly improving the electrolyte absorption rate [24]. Wen. et al. showed in experiments that it is feasible to improve the mechanical properties of electrospun films by hot pressing [25].

This study used a facile method to synthesize benzimidazole-containing diamine monomer [26], introduce it into the polyimide main chain, and use solid intermolecular forces to enhance the thermal and mechanical properties of the polymer [17,18,19]. The electrospinning method was used to make the polymer into a nanofiber film with a controllable thickness, and the mechanical properties of the nanofiber film were improved by hot pressing.

## 2. Materials and Methods

### 2.1. Materials

All reagents used in this study were of ACS reagent grade and were used without purification. 4-Aminobenzoic acid (PABA) and 3,3′-diaminobenzidine (DAB) were purchased from Sigma-Aldrich (St. Louis, MO, USA). Polyphosphoric acid (PPA, 84 wt%), phosphorus (V) oxide (P_2_O_5_), N, N-dimethylacetamide (DMAc), and dimethylsulfoxide (DMSO) were obtained from Acros Organics (Thermo Fisher Scientific, Waltham, MA, USA). 4,4′-Oxydiphthalic anhydride (ODPA) was from Alfa Aesar, Thermo Fisher Scientific Co. (Alfa Aesar, Thermo Fisher Scientific Co., Waltham, MA, USA). Sodium bicarbonate and methanol were from Duksan Pure Chemicals Co. (Duksan Pure Chemicals Co., Ansan, South Korea). Both DMAc and dimethyl DMSO were purified by stirring overnight in the presence of calcium hydride to remove water, followed by distillation under reduced pressure.

The weight of the active material is between 14.4 mg and 15.0 mg, its weight fraction is 93.5%, and it is loaded on an aluminum foil with a diameter of 12.8 mm. The weight of the nanofiber film material after being cut into a diameter of 17 mm is between 0.80 and 0.86 mg.

### 2.2. Methods

#### 2.2.1. Synthesis of 6,6′-bis[2-(4-Aminophenyl)Benzimidazole] (BAPBI)

**Figure 1:**
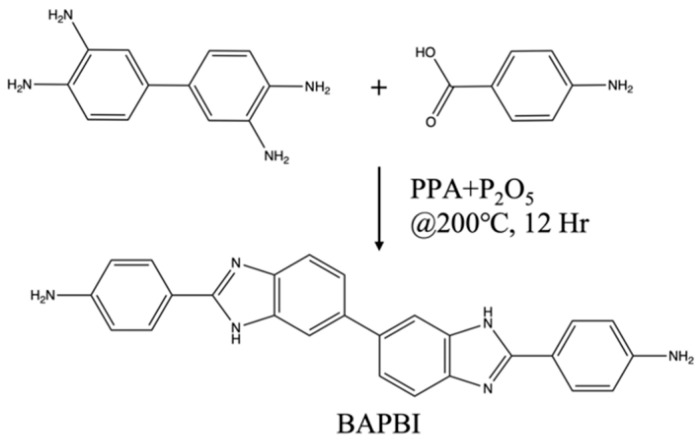
Schematic diagram of BAPBI synthesis.

Mix and stir 40 g of polyphosphoric acid and 5 g of phosphorus pentoxide until dissolved. Then, add p-aminobenzoic acid and 3,3′-diaminobenzidine and mix in a molar ratio of 2:1, stirring for 12 h at 200 °C. The reacted product was poured into 10 wt% sodium carbonate in a deionized aqueous solution to precipitate, and the precipitate was collected, washed with water, and then dried. The dried precipitate was dissolved in methanol, the filtrate was collected, and excess methanol was removed by vacuum drying to obtain the yellow product BAPBI (90% yield). The synthetic scheme is presented in Figure 1.

#### 2.2.2. Preparation of BI-PI Polymer

**Figure 2:**
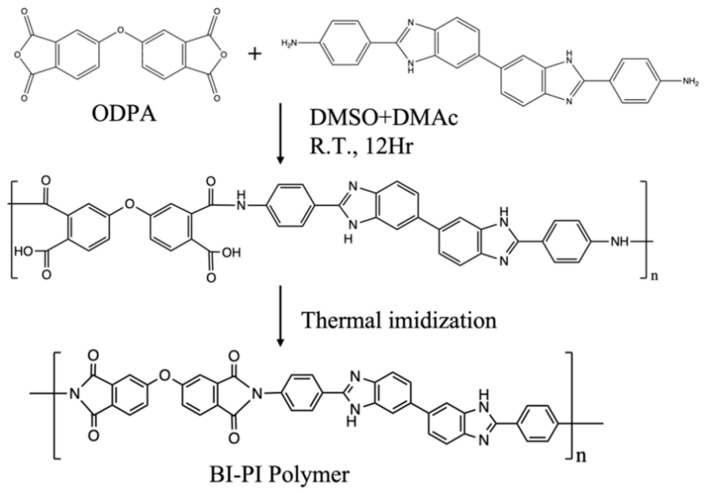
Schematic diagram of BI-PI preparation.

BI-PI was synthesized using a typical two-step method. BAPBI (1.8758 g, 4.5 mmol) was fully dissolved in 17.5 mL of DMSO and DMAc (volume ratio = 1:1) solution, and then ODPA (1.3980 g, 4.5 mmol) was slowly added, and the stirring continuously for 12 h in N_2_ atmosphere to obtain a homogeneous and viscous PAA solution with a solid content of 15.4 wt%. Electrospinning was then performed using a syringe with a spinneret having an inner diameter of 0.686 mm (19 G) and an applied voltage of 20 KV. PAA was then injected at a 0.01 mL min^−1^ rate, and the distance between the needle tip and the collector was 20 cm. In the atmosphere, nonwoven PAA nanofiber membranes were subjected to a continuous heating program (120, 200, 250, and 300 °C for 2 h each, 350, and 400 °C for 1 h each). Eventually, the imidized BI-PI nonwoven PAA was hot-pressed at 200 °C and 50 kgf cm^−2^ for 30 min. The synthetic scheme is presented in Figure 2.

### 2.3. Characterization

Structural identification, thermal properties, and porosity have been detailed in previous reports [26]. The surface morphology of the fibers was observed using field emission electron microscopy (SEM, JSM-6701F, JEOL, Tokyo, Japan). Thermogravimetric analysis (TGA, TGA 4000, Perkin Elmer, Washington, MA, USA) was used to detect the thermal degradation temperature of the polymer, which was raised from room temperature to 800 °C at 10 °C min^−1^ under a nitrogen atmosphere. A dynamic mechanical analysis instrument (DMA, TA Q 800, TA Instruments, New Castle, DE, USA) was operated at 1 Hz from 100 °C to 450 °C at 5 °C min^−1^ in a nitrogen atmosphere. A mercury porosimeter was used to measure the film’s porosity and pore size distribution (AutoPore^®^ IV 9520, Micromeritics, Norcross, GA, USA). A universal tensile machine (Al-7000-S, Gotech testing machines, Inc., Taichung, Taiwan) was used to measure the mechanical properties, and the test plates were cut into 150 mm × 5 mm and tested at a tensile rate of 12.5 mm min^−1^. The electrolyte absorptivity was measured by immersing the film in the LIB electrolyte for 30 min and calculated according to Equation (1) [27]:

(1) $$Electrolyte\;uptake=\frac{W_{1}-W_{0}}{W_{0}}\times 100\%$$

where *W*_0_ is the dry separator and *W*_1_ is the soaked one.

### 2.4. Electrochemistry Test

The separator material was cut into a circle with a diameter of 16 mm, soaked in the electrolyte (1 M LiPF_6_ in ethylene carbonate (EC)/ethyl methyl carbonate (EMC)/dimethyl carbonate (DMC) (1:1:1 wt%) + 1% vinylene carbonate (VC)) for 1 h, and placed in a glove box. The soaked separator material was sandwiched between two metal lithium sheets (Li/Separator/Li) with a diameter of 15.8 mm and assembled into a coin cell (CR2032). The AC impedance spectrum was obtained, and the open circuit voltage was measured from an electrochemical system (CHI600, CHI instrument, Inc., Austin, TX, USA). The alternating voltage amplitude was set at 10 mV, and the scanning frequency was 1 Hz–100 KHz to evaluate the electrochemical performance of the separator material. The ionic conductivity of the separator was estimated according to Equation (2), adopting bulk resistance (Rb) as the resistance, which was obtained from the EIS Nyquist equation [28]:

(2) $$\sigma =\frac{d}{R_{b}\times A}$$

where σ (mS·cm^−1^), Rb is the resistance in the high-frequency region, and d (cm) and A (cm^2^) are the ionic conductivity, membrane thickness, and membrane area, respectively. The diaphragm soaked in the electrolyte, lithium, and stainless steel (Li/Separator/SS) was assembled into CR2032. The linear sweeping voltage (LSV) method was performed at a scan rate of 10 mV s^−1^ at a voltage between 3 V and 5 V.

Half-cell tests of the LIB were performed by assembling lithium, separator, and LiFePO_4_ into a coin cell (CR2032). All battery materials were obtained from Ubiq Tech. Co., Ltd., Taoyuan, Taiwan. Stirring mixtures of lithium iron phosphate powder, PVDF, conductive carbon black (Super P), and carbon nanotubes with a weight ratio of 93.5:3:2.5:1 until uniform, coat them on aluminum foil and roll them after drying. The rolled electrode and lithium were cut into an electrode sheet with a diameter of 12.50 mm. The separator synthesized in the experiment was cut into a separator with a diameter of 17.00 mm by a knife and evaluated by a battery test device (CT2001, Lanhe, Wuhan, China). The separator’s performance in LIBs was compared with a commercial separator (Celgard H1612, Celgard, NC, USA). The voltage window was between 2.5 to 3.8 V, the rate test was set between 0.1 C and 2 C, and the cycle test was performed at 0.1 C.

## 3. Results

### 3.1. Properties of Electrospun BI-PI Separator

**Figure 3:**
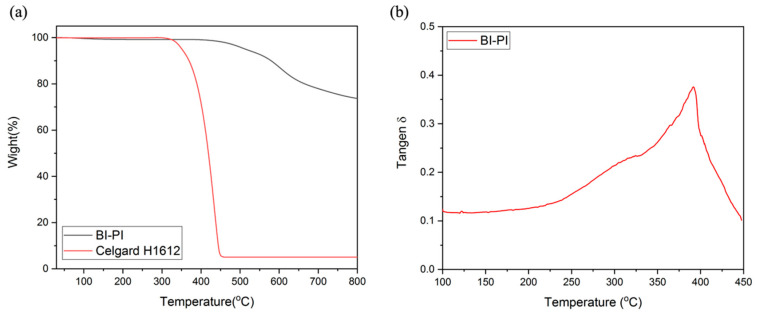
(**a**) TGA thermograms of BI-PI and Celgard H1612 in nitrogen. (**b**) DMA thermogram of BI-PI.

The thermogravimetric analysis in N_2_ (Figure 3a) demonstrates that BI-PI has four stages of weight loss at 120 °C, 500 °C, 600 °C, and 700 °C, respectively, and the Celgard film has only a single stage of weight loss at about 400 ℃. Figure 3a illustrates that BI-PI has excellent thermal stability Td5% up to 527 °C because of many aromatic structures in the main chain [29]. Compared with the Celgard H1612 polyolefin separator, BI-PI has high thermal stability. It is worth noting that the residual weight of BI-PI is as high as 73.69% at 800 °C, which is mainly due to the small proportion of imide functional groups and ether groups in the main chain of BI-PI [30]. In Figure 3b, it can be observed that there are two corresponding peaks of Tan δ, 395 °C, and 303 °C, respectively, and the main chain contains a soft segment, and the hard segment is the main reason for the two peaks of Tan δ. In the amorphous region of the polymer, the rigidity of the hard segment is relatively high, and it is not easy to move, while the soft segment is the opposite. The peak at 395 °C corresponds to the hard segment.

BI-PI has a slight weight loss at 120 °C, which is attributed to the adsorption on the surface of the electrospun fiber. The breaking of the imide ring and ether group between 500 °C and 600 °C causes weight loss. In this stage, gases such as CO, CO_2_, NH_3_, NO, and NO_2_ will release in the section, and the temperature at 700 °C is mainly caused by cracking on the benzene ring and imidazole ring [31,32]. The Celgard film material is a polyolefin, and at 400 °C, the bonding in the main chain is cracked, and C_2_H_4_ will be released.

**Figure 4:**
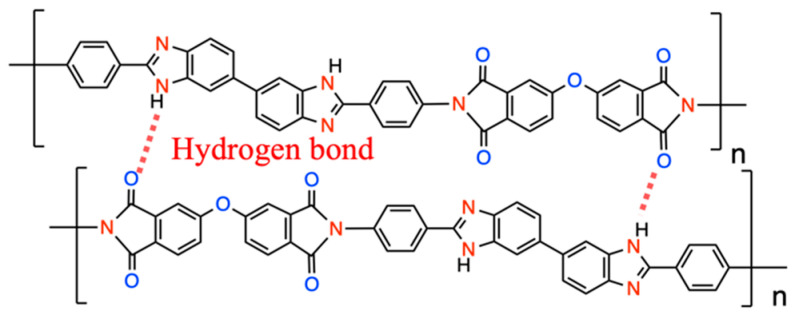
Schematic diagram of intermolecular H-bonding of BI-PI.

The formation of intermolecular hydrogen bonding (H-bonding) between imidazole and the imide group’s carbonyl also reinforces the main chains’ rigidity, as shown in Figure 4 [33]. It can be seen that BI-PI has good thermal properties and can withstand the high temperature of 300 °C without softening, which is sufficient in the application of LIBs.

The peak at 303 °C is because the backbone of the molecular chain contains a highly polar group, which may have a strong interaction with small molecules (e.g., residual solvent or water), thereby affecting the movement of the molecular chain and causing the relaxation temperature to occur in advance [34]. There will be a Tg as high as 395 °C because the main chain contains a large number of aromatic rings, high rigidity of the molecular chain, and hydrogen bonds between imidazole and the carbonyl on the imide group [33,34,35], as shown in Figure 4, make it difficult to increase the movement of polymer molecular chains [17,33]

Table 1 lists various properties of BI-PI film and Celgard H1612 and compares them with commercial Celgard H1612 separators. The electrolyte is the medium for transporting ions in the battery, and the absorption rate of the electrolyte significantly affects the ion transmission, thereby affecting the rate of performance and internal resistance of the battery [34]. BI-PI has a high porosity of 73% under the electrospinning process, which provides more space to accommodate the electrolyte. Because the operating voltage window of LIBs is much higher than the range that the aqueous electrolyte can tolerate, organic ester solvents are usually used (e.g., Ethylene carbonate, etc.) to dissolve lithium salts. The main chain contains many polar functional groups (e.g., N-H), which can interact with the electrolyte’s organic solvent and produce a strong affinity—and this is why the electrolyte absorption reaches as high as 1454%. The contact angle test results of the electrolyte and the film are shown in Figure S1. The BI-PI separator’s high electrolyte absorption rate and high electrolyte affinity are the main reasons for its nearly 20 times higher ion conductivity than commercial separators [36]. The EIS test results of the SS/Separator/SS system are shown in Figure S2.

It is generally believed that LIBs separators must have some dimensional stability when subjected to external stress [6,37]. The battery cells will be subjected to continuous curling stress during assembly. To avoid the short circuit originating from the separator displacement resulting from pressure, the displacement of the diaphragm must be controlled within less than 2% under a stress of 1000 Psi (6.89 MPa). Electrospun membranes have high porosity and can accommodate many electrolytes. On the contrary, this will also reduce the integrity of the mechanical structure, and the mechanical properties are generally poor [38]. The mechanical property test results are listed in Table 2, the tensile strength of the hot-pressed film increased from 10.92 MPa to 51.15 MPa, and the dimensional stability under stress also increased. SEM images showed that the fiber morphology also changed after hot pressing. It is generally considered that when the temperature is higher than 100 °C degrees Celsius, the intermolecular hydrogen bond force will fail, and the molecular chain will move more freely. In this experiment, the hot-pressing temperature reached 200 °C, and a substantial stress of 50 Kgf was continuously applied for 30 min. It will cause the molecular chains of BI-PI polymers to be continuously affected by high temperatures and intense stress so that the hydrogen bond force between the chains will fail, and the molecular chains will move and slide more easily. Therefore, the molecular chains can be fully rearranged, and the intermolecular force (hydrogen bond force) is more compact after the temperature is cooled. The molecular chains are rearranged under hot pressing, resulting in more physical crosslinking sites than before hot pressing, increasing the force between electrospun fibers and significantly improving mechanical properties. This phenomenon is similar to the sintering process.

**Figure 5:**
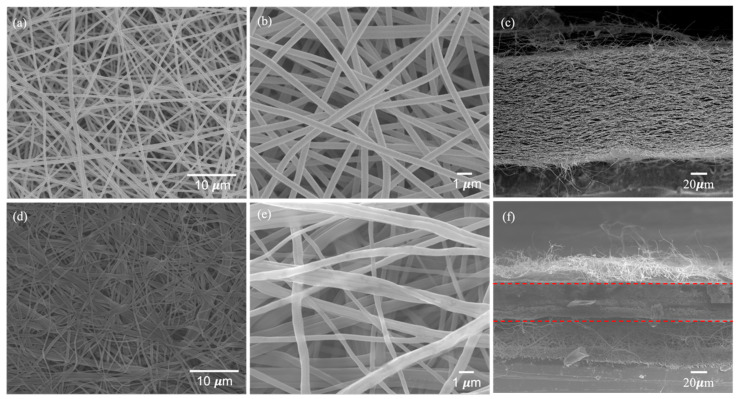
Morphologies of non-hot-pressed BI-PI separator membrane (**a**,**b**) surface, (**c**) cross-section type, hot-pressed one (**d**,**e**) surface, and (**f**) cross-section.

The SEM images in Figure 5a,b,d,e show the film’s surface morphology before and after hot pressing, and Figure 5c,f illustrate the cross-sectional shape of the film before and after hot pressing. Figure 5a,b demonstrate that the non-hot-pressed fibers of the film are independent and do not stick to each other. Figure 5c shows many pores between the fiber layers, and the fiber stacking distance is significant. After the hot-pressing process, the SEM image shows a completely different surface morphology from that before hot pressing. Figure 5d shows that the fibers oriented in the same direction are in contact with each other and stick together. Figure 5e shows the fibers oriented in different directions. There is also a stick at the intersection. The cross-sectional image of Figure 5f shows that the fibers are tightly packed, significantly reducing the gap between the fiber layers, and there is also a sticking phenomenon. These may be due to the pseudo-burning phenomenon caused by high temperatures and high-pressure stress at 200 °C for a long time, which leads to the easy transferring of stress between fibers and improves the mechanical properties of the electrospun film [25]. Figure S3 is the stress-strain diagram of the test.

**Figure 6:**
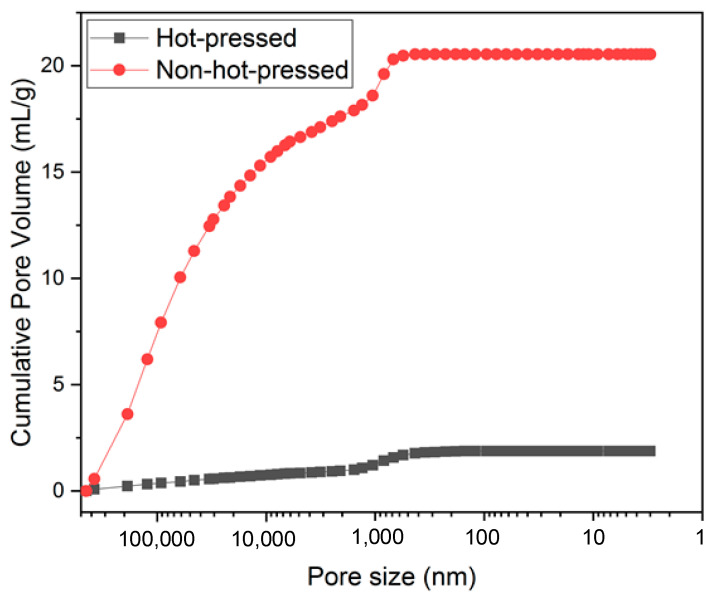
Cumulative pore volume distribution of films before and after hot pressing by mercury porosimetry.

The mercury porosimeter data in Figure 6 shows that the pore volume before hot pressing was 20.5 mL g^−1^, and the pore volume decreased to 1.9 mL g^−1^ after hot pressing. There is a 91% shrinkage of volume. Pore volume higher than 5000 nm decreased by 16.4 to 0.8 mL g^−1^, but the pore volume around 1000 nm did not change much from 2.0 to 0.66 mL g^−1^. It is because the types of pores in the electrospun film are mainly divided into two types: the pores formed by cross-stacking fibers in intercept directions, and this type of pore is about 1000 nm. Voids created between stacked layers can be larger than 5000 nm. The volume of voids reduced by the hot-pressing process belongs to the second type because the cross-stacking between fibers is more compact after hot pressing, significantly reducing the gap between the stacked layers (Figure 5c,f), which helps to reduce the film thickness. The separator has no electrochemical capacity contribution for batteries and will generate a specific internal resistance. Reducing the thickness of the separator helps to increase the volumetric energy density of the battery and reduce the internal resistance of the battery. Therefore, the proportion of the separator in the battery composition must be reduced to an appropriate thickness to improve the battery’s energy efficiency while maintaining the battery pack’s safe operation [37].

### 3.2. Application of BI-PI as Separators of LIBs

**Figure 7:**
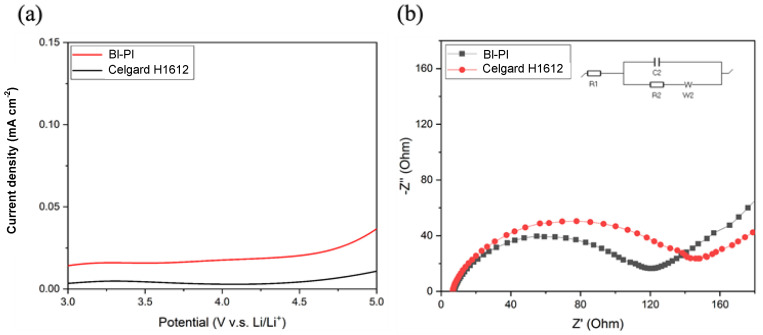
(**a**) LSV curves of Li/Separator/SS measured at a rate of 10 mV s^−1^ in the range of 3 V to 5 V (**b**) Nyquist plots obtained under Li/Separator/Li system.

Figure 7a demonstrates the LSV sweep curves of BI-PI and Celgard H1612 from 3 V to 5 V (relative to the Li+/Li electrode). All LSV curves have a low background current at potentials below 4.5 V and a slight increase in current beyond 4.5 V due to the reaction between the stainless-steel electrodes and the electrolyte diaphragm [36]. It can be seen that BI-PI has excellent chemical stability in the electrolyte of LIBs.

The interfacial charge transfer resistance (R_in_) between Li and the electrolyte layer is an important parameter that responds to the kinetics of electrochemical reactions [39]. R_in_ is closely related to the interface stability between the electrolyte layer and Li electrode, which will affect the cycle stability and rate performance of LIBs. Therefore, the compatibility of the membrane after absorbing the electrolyte with the lithium electrode can be studied by testing the EIS of the symmetric battery system (Li/electrolyte-absorbing membrane/Li). Figure 7b shows the Nyquist plots of BI-PI and Celgard H1612. The semicircle is present in all EIS spectra, illustrating the presence of Rin at the Li metal-electrolyte interface. From the EIS spectrum of Figure 7b, it can be found that the Rin of the BI-PI separator is 120 Ω smaller than the Rin 143 Ω of the Celgard H1612 separator. It is attributed to the BI-PI separator’s higher porosity and better electrolyte wettability. The pyrrole nitrogen of BAPBI on the main chain will create many defects at the lithium storage active sites [40]. In addition, the electron-rich nature of the imidazole ring interacts strongly with lithium ions [41,42]. These results make it easier for lithium ions to transport at the interface between the lithium electrode and the electrolyte layer, reducing the battery’s internal resistance and improving its rate performance.

**Figure 8:**
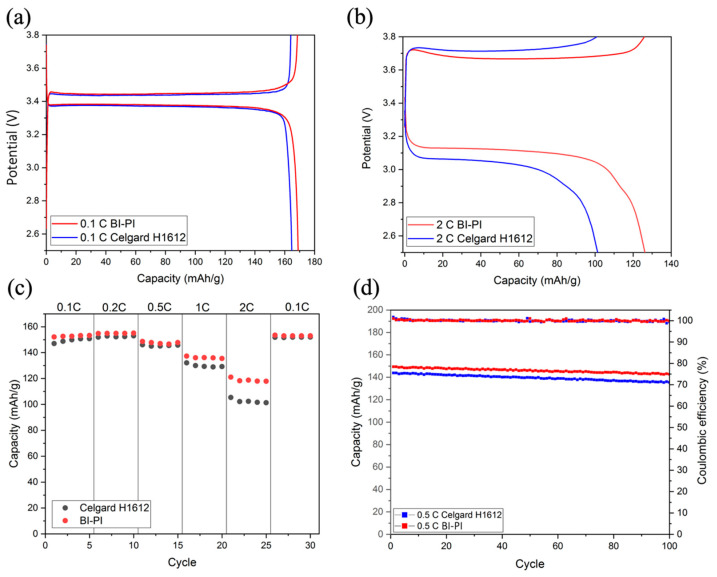
Charge-discharge curves of LiFePO_4_/Li half-cell system at (**a**) the first cycle at 0.1 C, (**b**) the 21st cycle at 2.0 C, (**c**) capacity at different current densities, (**d**) cycle stability test at 0.5 C.

Battery performance testing can comprehensively evaluate the effectiveness of separators applied to LIBs. By testing in a Li/LiFePO_4_ half-cell, the BI-PI separator’s cycle life and rate performance can be evaluated and compared with the commercial separator Celgard H1612. Figure 8a shows a Li-ion battery’s first charge and discharge curves at a low current density of 0.1 C. The results show that both BI-PI and Celgard H1612 have a stable charge-discharge form, and the capacity of BI-PI (169 mAh/g) is slightly higher than that of Celgard H1612 (166 mAh/g), but overall they are similar. However, when the current density is increased above 1 C, the influence of the separator on the battery performance is significant.

Figure 8b shows the 21st charge-discharge curve of the Li-ion battery at a high current density of 2.0 C. It can be found that when the current density increases, the discharge capacity of BI-PI is 126 mAh g^−1^, which is significantly higher than that of the commercial separator (101 mAh g^−1^). It is because the BI-PI nanofiber film has high porosity, high ion conductivity, and low charge transfer resistance.

The rate of performance of the battery is charged and discharged at various current densities between 0.1 C and 2 C, and the discharge results of the rate of performance are shown in Figure 8c. At low current densities, the capacity of the two separators was similar. However, when the current density was increased above 1 C, the BI-PI nanofiber membrane gradually widened the capacity gap with commercial thin films, taking advantage of low transfer resistance, high electrolyte absorption, and high ionic conductivity. It can be seen that the BI-PI nanofiber film has an excellent rate of performance at a high current density.

Cycling stability is an essential parameter for evaluating battery life. BI-PI and Celgard H1612 were activated for 5 cycles at a current density of 0.5 C and then charged and discharged 100 times. Figure 8d shows the cycle test results. It can be seen that both separators exhibit good cycle stability. After 100 cycles, the capacity retention rate of BI-PI is 96%, and that of Celgard H1612 is 94%. BI-PI is slightly higher than the commercial separator, which may be due to the high electrolyte absorption, excellent interfacial compatibility, and high ionic conductivity in the lithium anode [43].

Summarizing the above results and discussions, the molecularly designed BI-PI has an excellent performance in thermal properties and successfully improved the mechanical properties of the nanofiber film through the hot-pressing process. The film has high electrolyte wettability, a high electrolyte absorption rate, and good rate performance at high current density. The separator has been proven suitable as a high-performance supercapacitor in previous work [26]. The results and discussion in this study show that the separator is also suitable for high-performance LIBs. In the future, we will further enhance the lithium dendrite growth in the separator to improve LIBs’ performance and life cycles.

## 4. Conclusions

In this study, the BI-PI nanofiber film was successfully prepared by electrospinning. It has excellent thermal stability with a Td5% temperature as high as 527 °C and a Tg as high as 303 °C, making it stable when the battery is overheated, causing no short circuit resulting from separator melting. The BI-PI nanofiber film is densified through the hot-pressing process, the mechanical strength is increased four times, and the dimensional stability is improved. The BI-PI film demonstrates good electrochemical stability from 3 V to 4.5 V. Due to the high porosity of 73% and a high electrolyte affinity, BI-PI film has a high electrolyte absorption rate of 1454% and a high ion conductivity of 2.02 mS cm^−1^. When applied to LIBs, it is found to have high cycle stability, excellent rate performance at high current density, and lower charge transfer resistance compared with commercial separator Celgard H1612. The above shows that polyimides containing benzimidazole in the main chain are promising separator materials for a new generation of high-performance LIBs.

## Figures and Tables

**Table 1:** Comparison of BI-PI electrospun film and commercial Celgard H1612 separator.

	BI-PI	Celgard H1612
Thickness (μm)	50	16
Porosity (%)	73	44
Electrolyte uptake (%)	1454	101
Ionic conductivity (mS cm^−1^)	2.02	0.105

**Table 2:** Mechanical properties of BI-PI NFMS.

	Tensile Modulus (MPa)	Tensile Strength (MPa)	Elongation under 1000 Psi Stress (%)
Non-hot-pressed	171 ± 5.77	10.92 ± 0.97	3.64 ± 0.21
Hot-pressed	763 ± 22.01	51.15 ± 1.61	0.78 ± 0.02

## Data Availability

Not applicable.

## Supplementary Materials

The following supporting information can be downloaded at https://www.mdpi.com/article/10.3390/polym15081954/s1, Figure S1: Contact angles of (a) Celgard H1612 (b) BI-PI with electrolytes; Figure S2: EIS curves obtained in SS/Separator/ SS system; Figure S3: Stress-strain curves of hot-pressed and non-hot-pressed films.
