# Supplementary material for: Studies on the Application of Polyimidobenzimidazole Based Nanofiber Material as the Separation Membrane of Lithium-Ion Battery
Source: Polymers (Basel). 2023 Apr 20;15(8):1954. doi: 10.3390/polym15081954 (PMC10140945; doi:10.3390/polym15081954)
Supplement: Supplementary file 1 [file polymers-15-01954-s001.zip › polymers-2304357-supplementary.pdf]

polymers MDPI

Supplementary Materials

Studies on the Application of Polyimidobenzimidazole Based Nanofiber Material as the Separation Membrane of Lithium-Ion Battery

Yu-Hsiang Lu [1,†], Yu-Chang Huang [2,†], Yen-Zen Wang [1,*] and Ko-Shan Ho [2,*]

1 Department of Chemical and Materials Engineering, National Yu-Lin University of Science & Technology, 123, Sec. 3, University Rd., Dou-Liu City Yu-Lin, 64301, Taiwan; jerrylu34@gmail.com
2 Department of Chemical and Materials Engineering, National Kaohsiung University of Science and Technology, 415, Chien-Kuo Road, Kaohsiung 80782, Taiwan; ych@nkust.edu.tw
* Correspondence: wangzen@yuntech.edu.tw (Y.-Z.W.); hks@nkust.edu.tw (K.-S.H.)
† These authors contributed equally to this work.

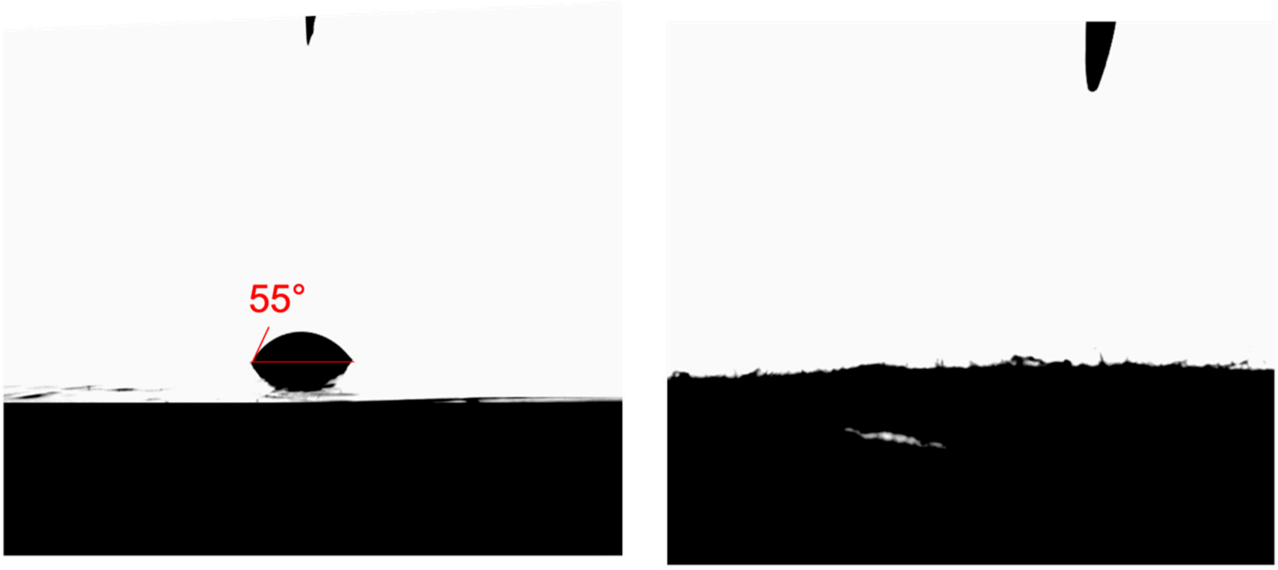

Figure S1. Contact angles of (a) Celgard H1612 (b) BI-PI with electrolytes.

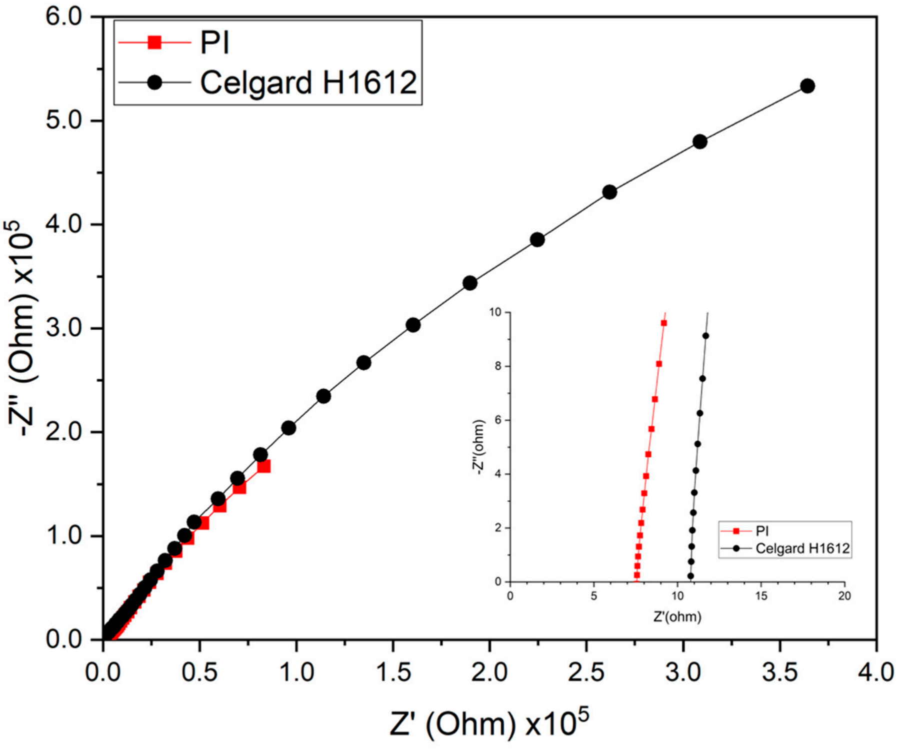

Figure S2. EIS curves obtained in SS/ Separator/ SS system.

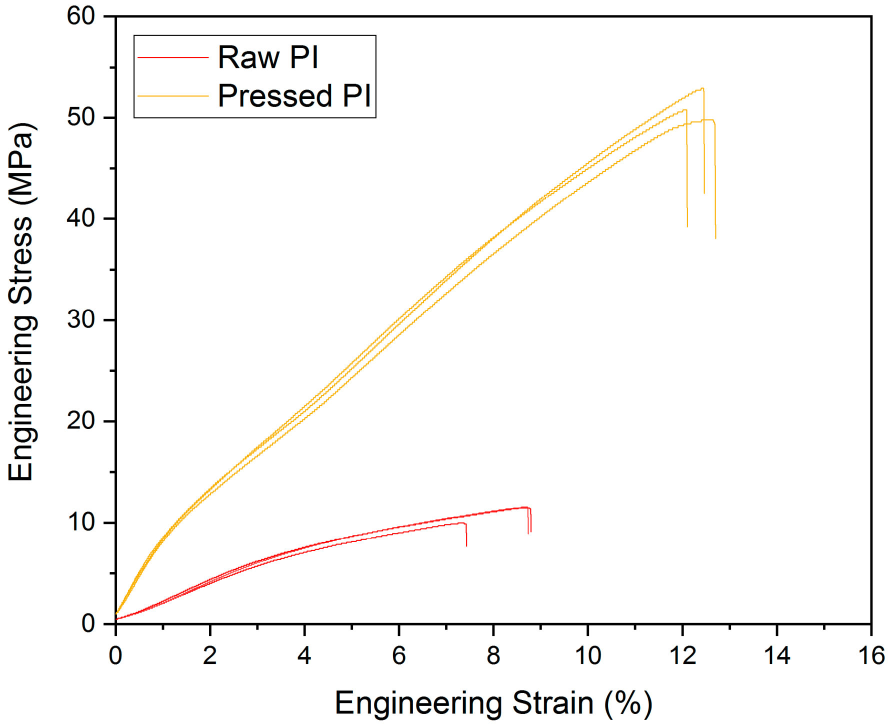

Figure S3. Stress-strain curves of hot-pressed and non hot-pressed films.
